# Integrating open-source technologies to build low-cost information systems for improved access to public health data

**DOI:** 10.1186/1476-072X-7-29

**Published:** 2008-06-09

**Authors:** Qian Yi, Richard E Hoskins, Elizabeth A Hillringhouse, Svend S Sorensen, Mark W Oberle, Sherrilynne S Fuller, James C Wallace

**Affiliations:** 1 Center for Public Health Informatics, University of Washington, Seattle, Washington, USA; 2 Washington State Department of Health, Olympia, Washington, USA

## Abstract

Effective public health practice relies on the availability of public health data sources and assessment tools to convey information to investigators, practitioners, policy makers, and the general public. Emerging communication technologies on the Internet can deliver all components of the "who, what, when, and where" quartet more quickly than ever with a potentially higher level of quality and assurance, using new analysis and visualization tools. Open-source software provides the opportunity to build low-cost information systems allowing health departments with modest resources access to modern data analysis and visualization tools. In this paper, we integrate open-source technologies and public health data to create a web information system which is accessible to a wide audience through the Internet. Our web application, "EpiVue," was tested using two public health datasets from the Washington State Cancer Registry and Washington State Center for Health Statistics. A third dataset shows the extensibility and scalability of EpiVue in displaying gender-based longevity statistics over a twenty-year interval for 3,143 United States counties. In addition to providing an integrated visualization framework, EpiVue's highly interactive web environment empowers users by allowing them to upload their own geospatial public health data in either comma-separated text files or MS Excel™ spreadsheet files and visualize the geospatial datasets with Google Maps™.

## Background

Information access is of critical importance for the practice of public health. Having timely, accurate and readily available information is essential to monitoring the health of communities and populations. Having access to public health data is essential to determining the association of environmental exposures to diseases, as well as measuring the progress and the efficacy of interventions. This information informs, educates and empowers people to develop a dialogue which results in effective programs and policies. It helps health authorities evaluate effectiveness, accessibility and quality of various public health services. It facilitates and supports data driven policy-making. The World Wide Web has enabled public health agencies with sufficient information technology resources to develop web-based information systems to extend their capacity for effectively using the data they collect. EpiQMS [1], developed by the state health department of Washington and the University of Washington, is currently used by health departments in Washington and Pennsylvania. It is implemented using a combination of proprietary operating systems and statistical packages. Many other information systems for analyzing, visualizing, and delivering public health data are implemented with proprietary software systems often beyond the reach of resource-constrained public health agencies. However, as open-source technologies continue to evolve, web-based information systems for collection, storage and analysis of public health data can be built quickly and efficiently with free open-source software and services. These open-source technologies can be shared with health agencies throughout the world and help resource-constrained public health agencies, including those in developing countries, utilize Internet resources with minimal development and support costs.

**Figure 1:**
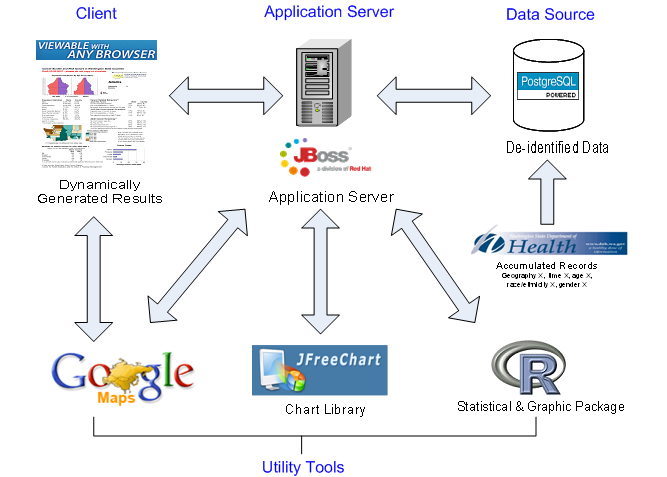
The EpiVue application architecture.

In this work, we explore the potential of open-source technologies by creating a web-based application framework for the visualization of public domain population health data. EpiVue, Epidemiologic Visual User Environment, is built exclusively with freely available open-source software. EpiVue components include PostgresSQL [2], a relational database for data storage, JBoss [3], a widely used J2EE Java application server, JFreeChart [4], an open-source Java chart library for use in applications, applets, servlets and Java Server Pages (JSP), the R [5] statistical computing and graphics toolkit and Google Maps™ [6] for interactive Geographic Information System (GIS) visualization. EpiVue is based on the Java programming language [7], enabling it to run on diverse computing platforms and operating systems. It is currently deployed in a conventional Linux operating system environment. EpiVue has been tested with a variety of popular web browsers and is specifically designed to require no additional application software or browser plug-ins which might limit its use. Figure 1 shows EpiVue's application architecture and the integration of the above open-source components.

Two reference datasets used in our EpiVue prototype are derived from accumulated Washington State Cancer Registry data [8] and death registry data from the Washington State Center for Health Statistics [9]. A third dataset was downloaded and incorporated into the EpiVue system from a life expectancy study covering a twenty-year interval for 3,143 United States counties [10]. Geographic data for United States state, county boundaries and ZIP code information are derived from ASCII formatted cartographic boundary files of Census 2000 of the United States Census Bureau [11].

## Results

**Figure 2:**
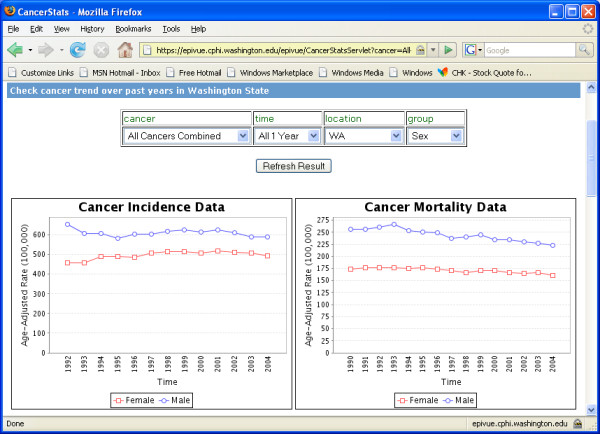
Line charts displaying the trends of cancer incidence and mortality age-adjusted rates/100,000 in Washington State over a multi-year period.

**Figure 3:**
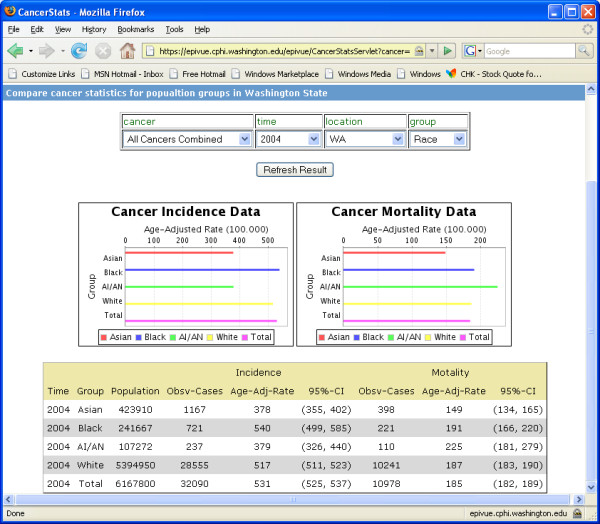
Bar charts and the corresponding data table comparing cancer incidence and mortality age-adjusted rates/100,000 for racial and ethnic groups in Washington State in 2004.

EpiVue data are queried through simple web selection controls. Results may be presented in tables, charts and maps. Figure 2 shows a pair of line charts displaying the trend of cancer incidence and mortality age-adjusted rates (per 100,000) over a 12-year period. Figure 3 shows a pair of bar charts and a table comparing cancer incidence and mortality age-adjusted rates (per 100,000) based on race and ethnicity. EpiVue currently uses JFreeChart [4] to generate charts as shown in Figure 2 and 3. However, the data analysis and graphics can also be implemented with R [5], an open-source language and environment for statistical computing and graphics. It provides a wide variety of statistical analysis and graphic capability comparable to commonly used commercial statistical software packages, such as SAS [28].

**Figure 4:**
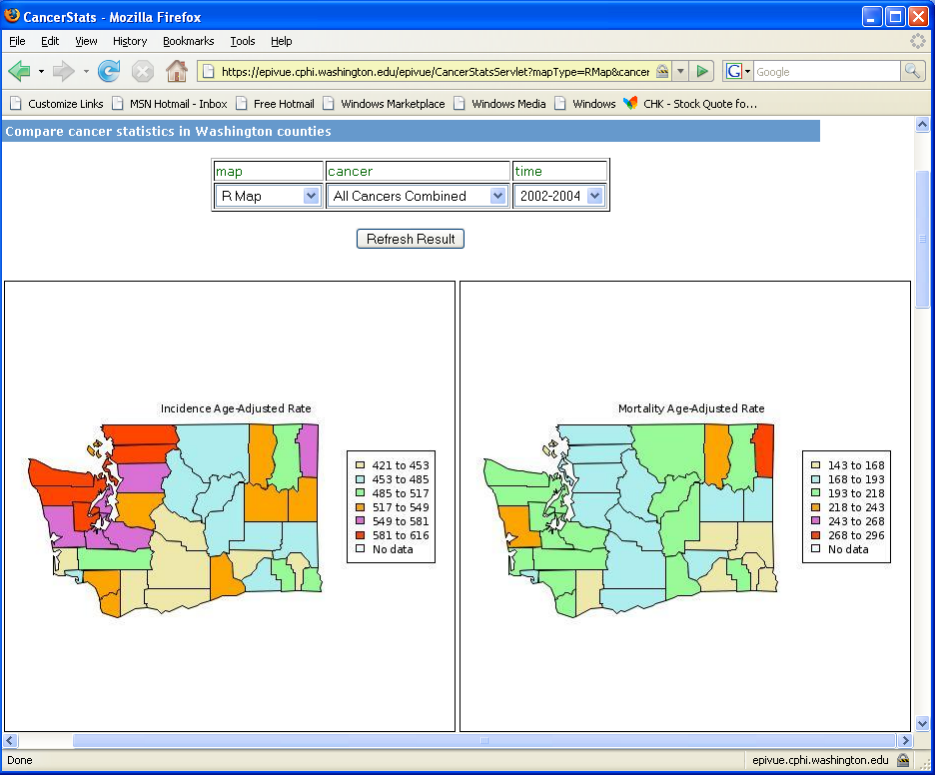
Side-by-side comparisons of incidence and mortality age-adjusted rates/100,000 among counties in Washington State presented in R maps.

**Figure 5:**
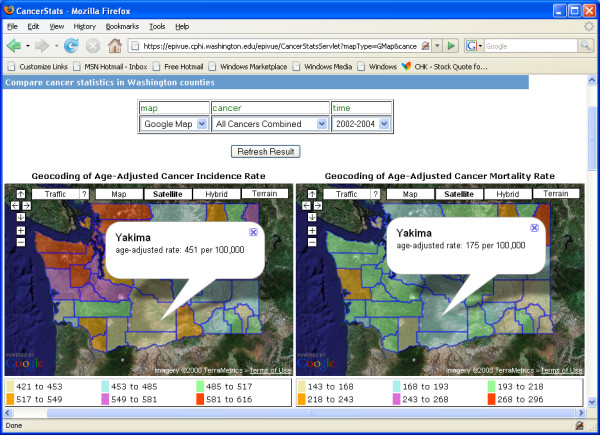
Side-by-side comparisons of incidence and mortality age-adjusted rates/100,000 among counties in Washington State presented in Google Maps™.

For visually analyzing epidemiological data to reveal geospatial trends that maybe hidden within charts and tables, EpiVue incorporates two open-source GIS utilities: the R language map utility [12] and the Google Maps™ Application Programming Interface (API) [6]. Figure 4 shows side-by-side Washington State county maps coded with cancer incidence and mortality age-adjusted rates (per 100,000) using the R map utility. EpiVue also uses Google Maps™ "mashups" to overlay Google Maps™ cartography data layers with deidentified public health data providing a second powerful and intuitive method for geospatial data visualization. Mashups are a new breed of web-based data integration applications which combine data from more than one source into a single integrated "virtual" application. Figure 5 displays side-by-side Washington State county colored polygon maps based on cancer incidence and mortality age-adjusted rates (per 100,000) using the Google Maps™ Keyhole Markup Language (KML) [25]. In comparison to the R language map utility, the Google Maps API is superior in terms of usability, integration, and coverage. In addition, the R map utility currently has very limited built-in geospatial coverage. However, the R map utility has the capability of displaying geospatial data entirely within the confines of a local health agency if deployed on a local server, thereby addressing privacy and security issues with identifiable data, while the Google Maps API requires client applications open to the public.

**Figure 6:**
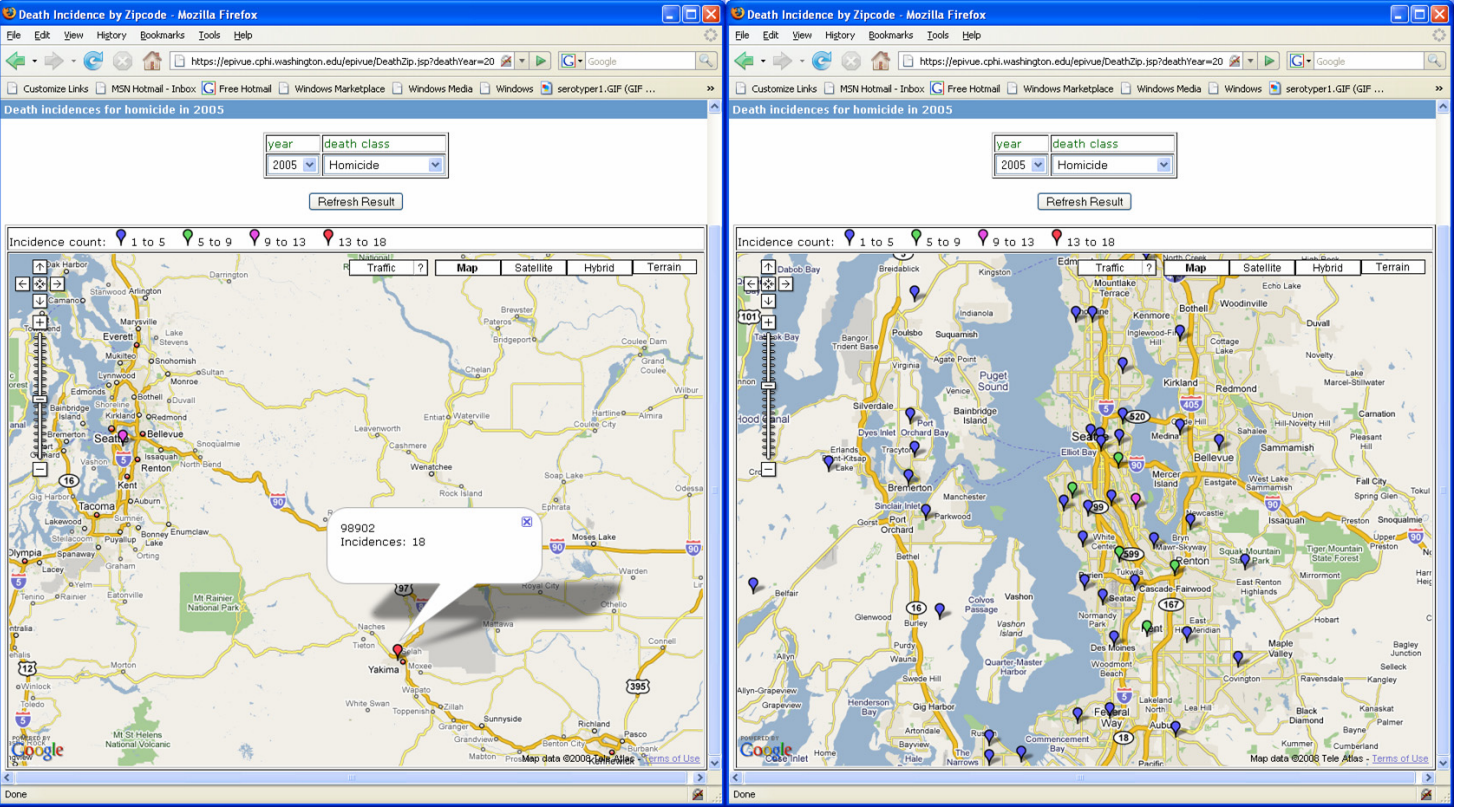
Maps generated with the Google Maps™ API showing death incidence data grouped by ZIP code and illustrating the EpiVue custom zoom feature.

EpiVue also utilizes AJAX (Asynchronous JavaScript and XML) support for Google Maps which exploits client side data caching to provide a highly responsive and interactive environment for visualizing geospatial data. Figure 6 shows death incidences from homicides in Washington State in 2005 grouped by ZIP codes which are represented by colored drop-shape markers. As users zoom into a region, more markers representing different levels of homicide incidences appear in the maps. This zooming capability provides a powerful way to display and manage a large amount of information in a relatively limited space.

**Figure 7:**
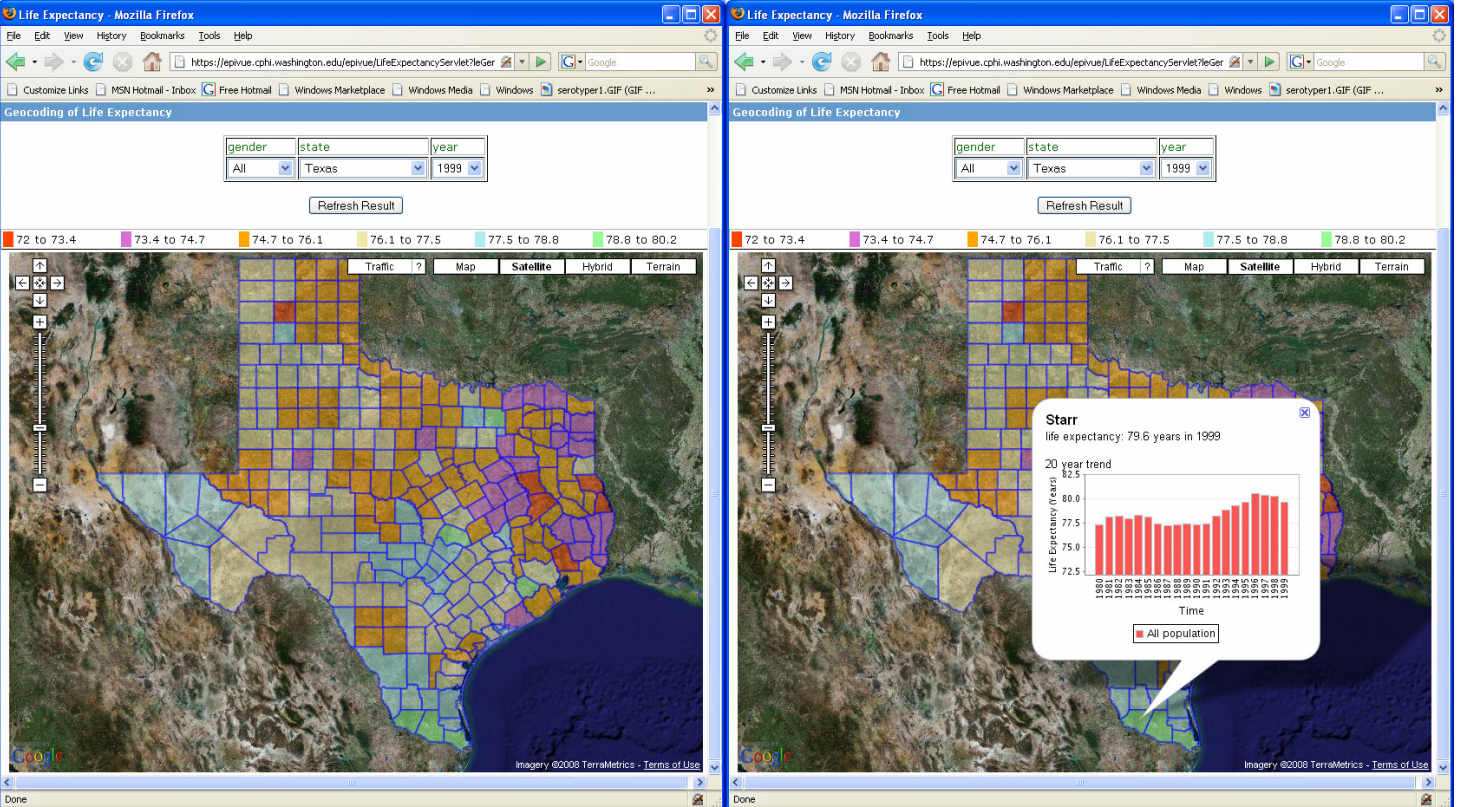
Left panel shows a Google Maps™ mashup with the life expectancy in 1999 for all genders in 254 Texas counties. Right panel is an embedded JFreeChart chart popup displaying the life expectancy trend from 1980 to 1999 for Starr County, Texas integrated into the Google Maps™ information window.

To test the scalability and performance of the EpiVue application, a dataset of life expectancy data covering all fifty U.S. states published by Murray et al. was downloaded and incorporated into EpiVue [10]. This dataset includes life expectancy statistics for 3,143 counties and the District of Columbia from 1980 to 1999. EpiVue currently limits users to viewing all counties in an individual state, the largest being Texas with 254 counties. Figure 7 shows a color-coded map based on the life expectancy for all genders in all Texas counties in 1999. When an individual county is clicked, an information window shows an embedded JfreeChart [4] bar chart displaying the trend of life expectancy from 1980 to 1999 for this county as shown in Figure 7. The ability to simultaneously display both spatial and temporal data in a highly interactive way represents an important extension of the Google Maps™ technique in its application to public health information.

**Figure 8:**
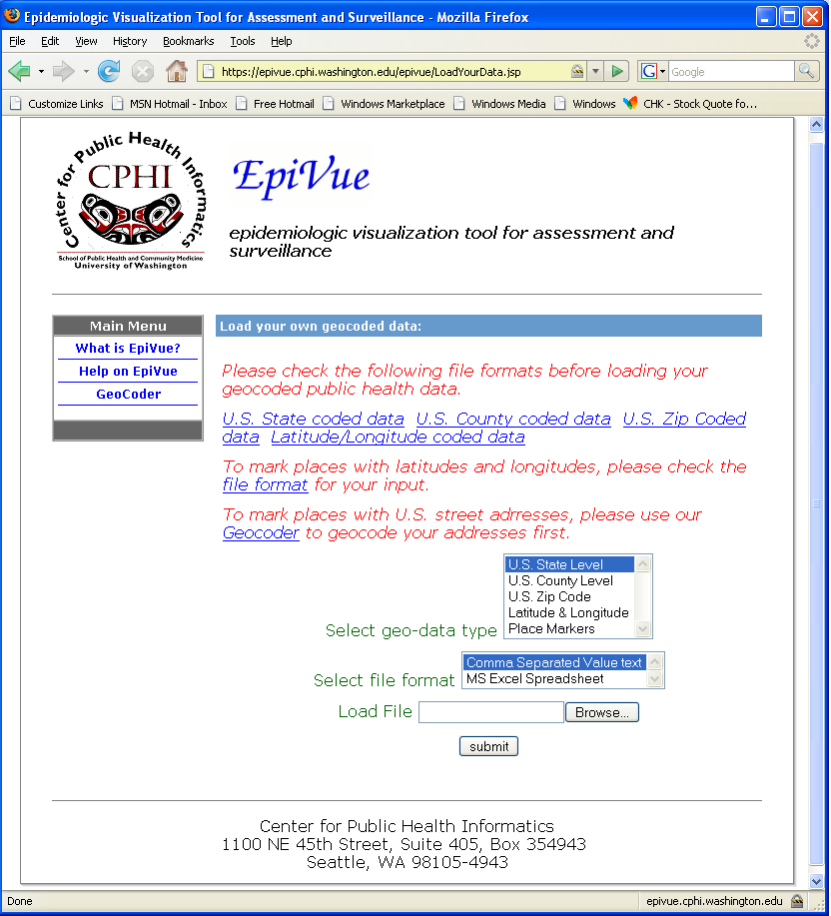
EpiVue interface to upload end-user data based on U.S. ZIP code, county, state or longitude and latitude.

**Figure 9:**
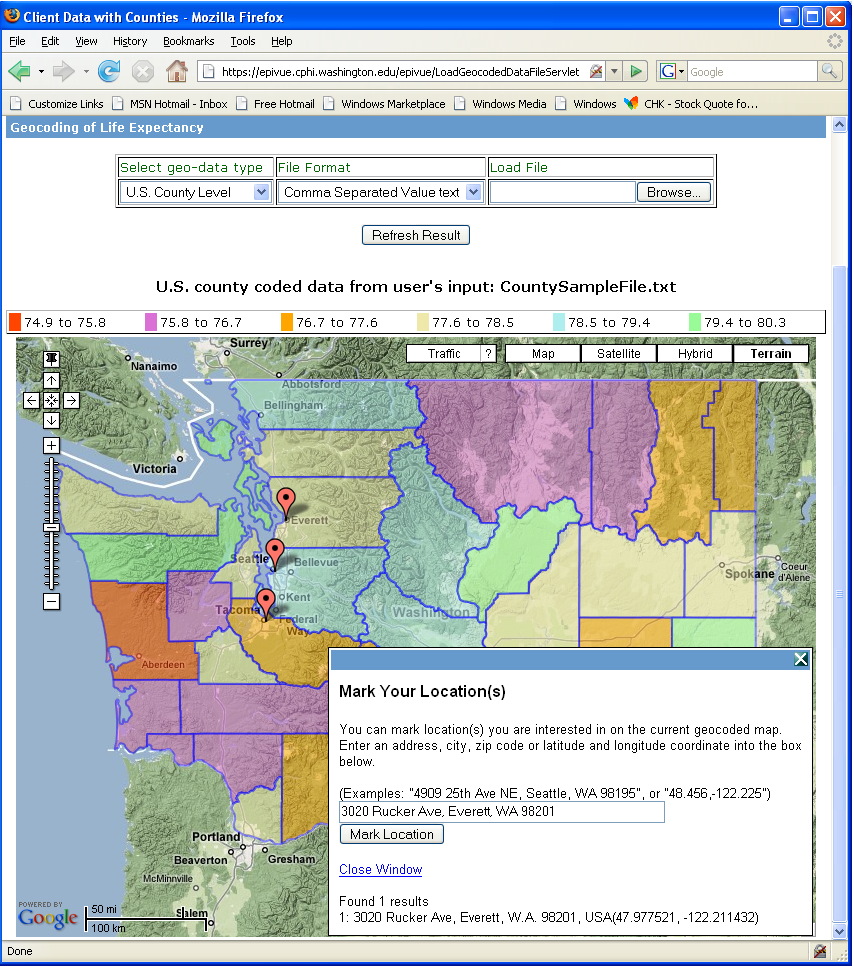
EpiVue interactive geocoder overlaid with users' geospatial data on Google Maps™.

EpiVue allows users to interactively upload and display their own geospatial data files as shown in Figure 8. Data files can be supplied in either comma-separated values (CSV) text format or Microsoft Excel™ format with values corresponding to the U.S. ZIP code, county, state or longitude and latitude. A built-in geocoder utilizing Google Maps geocoding services converts an uploaded file of street addresses to a corresponding longitude and latitude file. Furthermore, EpiVue also includes an interactive geocoder to be used along with users' geospatial data coded on Google Maps™ (see Figure 9). This feature allows the interactive addition of custom data on top of the existing geocoded data layer. Google Maps geocoding services currently cover 39 countries [26].

In summary, EpiVue's upload capability allows non-GIS-expert users to quickly visualize their geospatial data without additional support of GIS expertise or additional support of software plug-ins. It also provides a complete platform and roadmap for testing and modifying EpiVue for knowledgeable computer users seeking to deploy the EpiVue package locally. Example data files for file-uploading in each format are provided in Additional files section [see Additional files 1, 2, 3, 4, 5, 6, 7, 8, 9, 10, 11].

## Discussion

Access to public health data through the Internet has evolved rapidly in the past several years, especially in the United States, and many health agencies from the federal level through state and local health departments offer web access to public health data. Most of these applications are developed using proprietary software systems limited to presenting data in tabular format with no geospatial visualization capabilities. As public health awareness and interventions move beyond local, state and national boundaries towards a global health perspective, an increasing amount of public health data will need to be integrated and publicly accessible. The cost and complexity of implementing traditional proprietary solutions, however, will be a limiting factor for software system deployment in public health, especially for agencies with limited resources. For example, just the software cost alone for a web application built with Microsoft Windows server [27], Microsoft SQL database server [27], a commercial statistical package like SAS [28], and a commercial GIS package like ESRI [14] can easily reach tens of thousands of dollars. In contrast, the EpiVue application framework, composed exclusively of publicly available open-source technologies, can serve as a prototype for building low-cost public health visualization and assessment systems.

A recent study examining the prospects of using Internet platform GIS (or "web-GIS") with public health data indicated an overwhelming demand for the further integration of web-GIS into public health practice [13]. Many organizations want to use web-GIS but lack the resources and the expertise to build in-house web-GIS capacity. Web-GIS has the potential to become a standard method for displaying spatially referenced data and for empowering public health officials to employ spatial and temporal visualization in the dissemination of public health data. In comparison to other web-GIS packages, either proprietary packages such as ESRI's arcIMS [14], or open-source packages like MapServer [15], Google Maps™ has important advantages making it a candidate as a web-GIS platform for public health. These advantages include: (1) an easy to implement and ready-to-use free API for development mainly through JavaScript programming, (2) a large developer support community which makes it easy to find technical solutions and support online, (3) a generic browser interface requiring no additional software or plug-ins for most of modern browsers like Internet Explorer, Firefox, and Safari and (4) an extensive international user community who is already familiar with Google Maps™ and requires little, if any, additional training.

Challenges and limitations clearly exist in deploying public health data using web-GIS open-source technologies in terms of security and privacy. Google Maps™ in particular requires spatial data coordinates to traverse the Internet in order to utilize web-GIS capabilities. Still, a large body of valuable public health data is not limited by privacy and security constraints, such as the publicly available cumulative datasets [8-11] used in this study. Many researchers are already deploying web-GIS visualization in their applications in diverse public health arenas [16-18,29,30]. AEGIS [16] uses the Google Maps™ API to display simulated temporal and spatial alarms related to syndromic surveillance. HealthMap [17] pinpoints world-wide infectious disease outbreak information in a web-GIS interface using Google Maps™. The WhoIsSick website [18] combines an innovative social networking schema with Google Maps™ mashups for voluntary anonymous reporting of infectious disease symptoms.

Another limitation in using the Google Maps API for web-GIS is the size of the geocoded data sent to the Google Maps server for rendering on the client browser system. Based on our experience, the Google Maps server is able to render approximately 100,000 latitude/longitude boundary points per geocoded overlay. Although this is sufficient to display all 254 Texas county boundaries at full resolution in the life expectancy example above, it falls short of the approximately 150,000 points required to display all fifty states in the United States at full resolution using the 2000 U.S. Census data files. We were able to get around this limitation by sampling every third data point without significant compromises in performance or resolution. A larger issue in building low-cost web-GIS systems is the lack of freely available geographic data sets, particularly for use in underserved countries. Currently, the U.S. Census Bureau has made census related geographic data at levels of state, county, ZIP code, census block, and census tract available to the public through its web site [11]. Canada is also providing some quality geographic data at no cost for the public through GeoBase [31].

Interoperability with existing health data and software systems will be crucial if the use of web-GIS in public health is to gain acceptance among public health practitioners and the general public. EpiVue is specifically designed for simplicity and interoperability at both the web browser level and at the systems level to achieve broad end-user acceptance while at the same time promoting ease of implementation and support for local public health agency installations.

At the browser level, EpiVue runs on all common web browsers without additional plug-ins or local application software. Requiring the use of specific web browsers, or browser plug-ins, introduces complexity, incompatibility and security issues which can discourage widespread adoption of web applications. However, nothing in the EpiVue applications framework precludes the use of plug-ins. For example, open-source web-GIS tools such as Scalable Vector Graphics (SVG) [19] have already made their way into public health visualization applications [1,20]. SVG and other plug-in technologies such as Adobe Flash could be valuable additions to future versions of the EpiVue suite of open-source tools.

EpiVue's system architecture is designed for future growth and interoperability with other software systems and services. EpiVue's Java based application framework lends itself to integration with other Java applications such as AEGIS-CCT [16,21], an open-source software tool for creating simulated outbreak clusters in surveillance systems. The EpiVue JBoss server features built-in web services capabilities which can be used to link services to a larger public health computing grid. The R statistical language and graphics package included in the EpiVue application framework could be further exploited for its extensive capability of performing complex bio-statistical analysis [22]. R is an open-source alternative to proprietary statistical packages unavailable to resource-constrained health agencies.

EpiVue may be particularly well suited to public health disaster preparedness applications. The Google Maps™ API now includes traffic flow information for 30 major United States cities which could be exploited for monitoring traffic conditions in areas proximal to disasters. It also features a terrain data layer which could be used to show areas susceptible to flooding and water/well contamination in flood prone regions such as Washington State.

## Conclusion

We believe the EpiVue application framework can serve as a prototype for building low-cost public health visualization and assessment systems for resource-constrained environments. In the future, we envision integrating the EpiVue application framework with our public health knowledge management system, myPublicHealth [23], to create a comprehensive "dashboard" allowing public health officials to quickly assess the health of their local communities and respond rapidly to adverse public health events.

## Methods

EpiVue was designed using the conventional 4-tier J2EE architecture consisting of a data source tier using a PostgreSQL database and static files, a business tier utilizing Enterprise Java Beans (EJB), a web tier using servlets and Java Server Pages (JSP), and a generic browser client tier.

The three datasets used in this work were originally obtained in different formats. The Washington State cancer dataset [8] was in tab-delimited ASCII format, the Washington State death dataset [9] was in Xbase data file (.dbf) format, and the life expectancy dataset [10] was in Microsoft Excel spreadsheet format (.xls). Each of these files was parsed using Perl [24] programs with the following Perl utility packages: XBase.pm for .dbf files and Spreadsheet.pm for .xsl files. Parsed datasets were loaded into a PostgreSQL [2] relational database using the Perl DBI.pm module. All Perl modules mentioned above are freely available for download from CPAN [32]. The geographic data for state, county boundaries and ZIP codes were downloaded from U.S. Census 2000 by the U.S. Census Bureau [11]. The ZIP code geographic data were loaded into the PostgreSQL [2] database and each ZIP code is represented by its centroid latitude and longitude. County and state boundary data were stored in both the PostgreSQL database and static tab-delimited ASCII files.

The web tier and the business tier are components of the JBoss J2EE application server [3]. The EJB beans in the business tier interact with the database and perform all business queries. Servlets in the web tier process client requests then pass them to EJB beans and obtain the query results from EJB beans. Servlets also integrate various utility tools listed in Figure 1 to generate responses to client requests. Java Server Pages (JSP) in the web tier is used to present dynamic responses to clients.

The client tier is made up of static and dynamic html pages including forms, tables, charts, and embedded Javascripts with AJAX capability for Google Maps™. AJAX is a group of inter-related web development techniques used for creating interactive web applications. It is asynchronous, in that the extra data is requested from the server and loaded in the background without interfering with the display and behavior of an existing page. Most modern browsers, such as Internet Explorer, Firefox, and Safari, will work properly without any additional plug-ins. The state and county level polygon maps are implemented using Google Maps™ support for KML [25] or KMZ. The maps for ZIP codes or latitude/longitude data are implemented using AJAX programming with the Google Maps™ API.

EpiVue is accessible through its web site [33].

## Supplementary Material

Additional file 1 CountySampleFile.txt. U. S. county based geospatial public health data in comma separated value text format. Click here for file

Additional file 2 CountySampleFile.xls. U. S. county based geospatial public health data in Microsoft Excel spreadsheet file format. Click here for file

Additional file 3 ZipCodeSampleFile.txt. U. S. ZIP code based geospatial public health data in comma separated value text format. Click here for file

Additional file 4 ZipCodeSampleFile.xls. U. S. ZIP code based geospatial public health data in Microsoft Excel spreadsheet file format. Click here for file

Additional file 5 LatLngSampleFile.txt. Latitude/longitude based geospatial public health data in comma separated value text format. Click here for file

Additional file 6 LatLngSampleFile.xls. Latitude/longitude based geospatial public health data in Microsoft Excel spreadsheet file format. Click here for file

Additional file 7 StateSampleFile.txt. U. S. state based geospatial public health data in comma separated value text format. Click here for file

Additional file 8 StateSampleFile.xls. U. S. state based geospatial public health data in Microsoft Excel spreadsheet file format. Click here for file

Additional file 9 InputForGeocoderSample.txt. U. S. street address sample file in tab separated text file format. Click here for file

Additional file 10 InputForGeocoderSample.xls. U. S. street address sample file in Microsoft Excel spreadsheet file format. Click here for file

Additional file 11 PlaceMarkersSample.xml. locations with known latitudes/longitudes sample file in XML format. Click here for file
